# GEM-YOLO: A Lightweight and Real-Time RGBT Object Detector with Gated Multimodal Fusion

**DOI:** 10.3390/s26072035

**Published:** 2026-03-25

**Authors:** Lijuan Wang, Zuchao Bao, Dongming Lu

**Affiliations:** 1 School of Electronic and Optical Engineering, Nanjing University of Science and Technology ZiJin College, Nanjing 210023, China; 2 School of Electronic and Optical Engineering, Nanjing University of Science and Technology, Nanjing 210094, China

**Keywords:** multispectral object detection, infrared-visible fusion, small object detection, edge deployment, modality calibration

## Abstract

Red–Green–Blue–Thermal (RGBT) object detection is critical for robust all-weather perception. However, deploying dual-stream networks on resource-constrained edge devices is severely hindered by insufficiently adaptive multimodal fusion, the loss of small-object features during downsampling, and substantial computational overhead. To address these challenges, we propose GEM-YOLO, a real-time and lightweight RGBT detector. Specifically, an Adaptive Multimodal Gated Fusion Mechanism (GFM) is designed to dynamically calibrate modality weights and suppress noise. Furthermore, Space-to-Depth (SPD) convolutions are integrated into the backbone to achieve lossless downsampling, preventing the feature collapse of small targets. Finally, a lightweight Ghost-Neck is constructed using Ghost modules and GSConv to eliminate computational redundancy. Extensive experiments on the Forward-Looking Infrared (FLIR) and Multi-Modal Multispectral Fusion Dataset (M3FD) datasets demonstrate the effectiveness of the proposed method. With only 7.58 Giga Floating-Point Operations (GFLOPs) and 3.44 million parameters (M), GEM-YOLO reduces the computational cost by 18.6% relative to the dual-stream YOLOv11n baseline. Concurrently, it achieves competitive mean Average Precision at IoU = 0.5 (mAP@50) scores of 82.8% and 69.0% on FLIR and M3FD, respectively, with more evident gains on small-target localization. In practice, GEM-YOLO maintains competitive detection performance while keeping computational overhead low, making it promising for real-time multispectral perception on resource-constrained edge platforms.

## 1. Introduction

Object detection is a fundamental task in computer vision, serving as the perceptual cornerstone for numerous safety-critical applications, including autonomous driving, intelligent video surveillance, and search and rescue operations [1,2]. Over the past decade, deep learning-based detectors, especially the YOLO (You Only Look Once) series, have achieved strong performance under favorable illumination conditions by maintaining a good balance between detection accuracy and inference speed [3]. However, visible-light (RGB) cameras rely heavily on ambient lighting. Their performance drops markedly in challenging environments, such as complete darkness, dense fog, smoke, or strong glare, where visual cues may become weak or even unavailable [4]. To compensate for this limitation, thermal infrared (T) sensors have been introduced. Unlike RGB cameras, thermal sensors capture object emissivity rather than reflected light, making them less sensitive to illumination changes and more effective in smoke-obscured scenes. Nevertheless, thermal images usually contain less texture and no color information [5]. For this reason, RGBT object detection has attracted increasing attention, since it combines the complementary advantages of visible and thermal modalities to support more robust all-weather perception [6].

Despite the progress made in recent multispectral detection, developing an RGBT detector that is both accurate and practical for edge deployment remains difficult. In real applications, three problems are repeatedly encountered. First, multimodal fusion is often not sufficiently adaptive. Second, small-object features are easily weakened during repeated downsampling. Third, improving representation ability usually increases computational cost, which makes real-time deployment more difficult.

Among these issues, multimodal fusion is especially critical. Although RGB and thermal information are theoretically complementary, their usefulness is highly scene-dependent. In some cases, the two modalities support each other well, whereas in other cases, one branch may become unreliable and even interfere with fusion. Many existing methods adopt a dual-branch backbone and combine features using simple operations such as channel concatenation or element-wise addition [7]. These strategies are easy to implement and efficient in computation, but they implicitly assume that both modalities remain equally informative across different scenes.

This assumption is often too idealized. Under daytime conditions, RGB images usually provide richer texture and clearer structural details, while thermal images may suffer from thermal crossover, where the temperature contrast between the target and the background becomes weak. In such cases, the thermal branch may contribute little useful discrimination and may even introduce interference. At night, the situation is often reversed. RGB images degrade because of insufficient illumination, whereas thermal images can still preserve clearer target contours. As reported in [8], early fusion strategies are particularly vulnerable to noise propagation because irrelevant responses from one modality may be directly introduced into the shared feature space.

Simple concatenation is unable to distinguish reliable information from degraded responses. Once noisy features are mixed into the fusion process, they may weaken the contribution of more informative cues from the other modality and finally reduce detection performance [9]. Therefore, multimodal fusion should not remain fixed across all scenes. A more reasonable strategy is to adjust the contribution of each modality according to scene conditions and feature reliability. How to design such an adaptive yet efficient fusion mechanism is still an important problem in practical RGBT detection.

Another practical difficulty lies in the loss of small-object information during feature extraction. In real-world scenarios such as autonomous driving, distant or small targets—including pedestrians, motorcycles, and traffic signs—must be reliably detected to ensure safe operation. However, widely used multispectral datasets like FLIR [10] and M3FD [11] contain a large proportion of weak and small infrared targets embedded in complex backgrounds. Traditional Convolutional Neural Networks (CNNs), including the standard YOLOv8/v11 architectures, often use strided convolutions (e.g., stride = 2) to reduce spatial resolution. While this increases the receptive field, repeated downsampling may attenuate or obscure fine-grained features of small targets. The effective loss of information depends on factors such as kernel size, padding, pooling, and the nature of learned features. Sunkara et al. [12] identified this phenomenon as a major cause of low recall rates for small objects. Although some approaches attempt to mitigate this by adding high-resolution detection heads (e.g., P2 layer), such modifications significantly increase memory consumption (Video RAM, VRAM) and inference latency, making them unsuitable for embedded devices.

The third challenge lies in the high computational burden of RGBT detection on edge devices. In practical applications such as drones, embedded cameras, and mobile robots, the detector must maintain a careful balance between accuracy and efficiency. However, most RGBT detectors adopt a dual-stream backbone to process visible and thermal inputs separately, which usually increases both parameter count and computational cost compared with single-modal detectors. In many cases, the overall overhead is about 1.5 to 2 times higher.

Another issue comes from the network architecture itself. Standard YOLO-style designs still rely on relatively heavy modules in the neck, such as C3k2 or Bottleneck blocks, which introduce considerable computational redundancy. As pointed out by Han et al. in GhostNet [13], many feature maps generated by deep networks are in fact highly similar, meaning that part of the computation is redundant. A direct reduction in network depth, for example, by using a slimmed neck structure, may improve efficiency to some extent, but it often weakens feature aggregation and leads to noticeable accuracy degradation [14].

As a result, existing RGBT detectors still find it difficult to satisfy the real-time requirements of edge deployment. This problem becomes even more evident on resource-limited hardware platforms, such as the NVIDIA Jetson series, where practical runtime is constrained not only by FLOPs but also by memory access, operator efficiency, and hardware parallelism.

To better motivate the objective of this study, the main challenges discussed above can be concisely summarized as follows: (1) static multimodal fusion tends to propagate modality-specific noise when the reliability of RGB and thermal inputs varies across scenes; (2) conventional downsampling operations often cause irreversible loss of small-object features, which is particularly harmful for distant thermal targets; and (3) dual-stream RGB-T architectures usually introduce substantial computational overhead, making real-time deployment on resource-constrained edge platforms difficult.

To address these challenges, we propose GEM-YOLO, a lightweight and real-time RGBT object detector for edge-oriented multispectral perception. Rather than introducing entirely new standalone low-level operators, GEM-YOLO is designed as a task-oriented architectural framework that jointly considers adaptive multimodal fusion, small-object feature preservation, and computational efficiency under dual-stream constraints. The main contributions of this study are summarized as follows:

(a) Adaptive Multimodal Gated Fusion Mechanism (GFM): We design a lightweight global-context gating mechanism between the dual backbones to adaptively regulate the contributions of RGB and thermal features under different scene conditions. Instead of static fusion, GFM estimates complementary modality weights from globally pooled RGB–IR features through a compact Multi-Layer Perceptron (MLP) followed by Softmax normalization, enabling scene-adaptive modality calibration with negligible computational overhead.
(b) Feature-Preserving Backbone via SPD-Conv: To alleviate the “small object disappearance” problem, we redesign the backbone by incorporating Space-to-Depth (SPD) convolutions [12]. By replacing traditional strided convolutions with SPD blocks, spatial details are reorganized into the channel dimension, which helps preserve texture and contour features for small thermal targets. This feature-preserving downsampling design is beneficial for maintaining fine-grained target representations under repeated resolution reduction.
(c) Real-Time Lightweight Neck based on Ghost and Ghost-Shuffle Convolution (GSConv): We reconstruct the detector’s neck using Ghost Modules [13] and GSConv [14] to alleviate the computational bottleneck of dual-stream networks. By replacing redundant standard convolutions with more efficient operations, we reduce the computational cost to less than 9.0 GFLOPs—comparable to single-stream networks—while maintaining competitive feature representation capability.

The remainder of this paper is organized as follows: Section 2 reviews related work. Section 3 details the proposed GEM-YOLO architecture. Section 4 describes the experimental setup, including the datasets, evaluation metrics, and implementation details. Section 5 presents the experimental results and analysis on the FLIR and M3FD datasets, including comprehensive comparisons with state-of-the-art methods and detailed ablation studies. Section 6 provides the conclusion of this paper.

## 2. Related Work

### 2.1. RGBT Fusion Mechanisms: From Static to Dynamic

One of the main challenges in RGBT detection is effectively using complementary information from visible and thermal modalities. Fusion strategies have gradually developed from simple static operations to more adaptive nonlinear attention-based mechanisms.

Early deep learning-based approaches primarily adopted decision-level or feature-level fusion strategies, such as concatenation or element-wise addition [8]. Although computationally efficient, these static fusion schemes implicitly assume equal reliability across modalities. In practical scenarios, however, modality reliability varies significantly. Thermal sensors may suffer from crossover effects, while RGB images degrade under low illumination. As reported by Zhao et al. [15], static fusion inevitably propagates modality-specific noise into shared feature representations, thereby compromising detection robustness. Dual-stream architectures such as Dual-YOLO [9] improved feature extraction capacity but still lacked pixel-level or spatially adaptive fusion mechanisms.

To address this limitation, recent research has shifted toward dynamic calibration strategies, which is particularly crucial when processing strictly aligned target pairs in complex low-light environments, such as those introduced in the LLVIP benchmark [16]. Instead of relying on computationally expensive auxiliary branches, recent architectures focus on highly efficient cross-modal feature alignment. Approaches such as INSANet [17] and the multiscale confidence-aware fusion network proposed by Li et al. [18] employ advanced cross-modality attention mechanisms to capture long-range dependencies and dynamically enhance inter-modal correlations. More recently, MSDF-Mamba [19] explores State Space Models (SSM) to achieve linear-complexity sequence modeling for highly efficient multimodal fusion.

Despite their strong representational power, Transformer- and SSM-based methods introduce substantial computational burdens. Self-attention operations incur quadratic complexity $O(N^{2})$ with respect to token length, which limits scalability for high-resolution inputs. Although SSM-based methods reduce theoretical complexity, their hardware-level optimization on embedded GPUs (e.g., Jetson-class devices) remains less mature than conventional CNN operators.

In contrast, the proposed Gated Fusion Module (GFM) adopts a lightweight global-context gating mechanism to enable dynamic modality selection without heavy global attention operations. By replacing computationally expensive cross-attention with scene-level adaptive gating, GFM achieves dynamic feature calibration with negligible additional computational cost, making it more suitable for real-time edge deployment.

Representative RGB-T and multispectral fusion studies can be further divided into several typical directions. Early dual-stream RGB-T fusion architectures, such as RTFNet [20], demonstrated the effectiveness of combining visible and thermal representations within a two-branch framework. Subsequent detection-oriented methods, such as MBNet [21], explicitly addressed modality imbalance under different illumination conditions, while ProbEn [22] explored probabilistic ensembling of RGB and thermal detectors to improve robustness at the decision level. More recently, attention- and transformer-based methods, such as GAFF [23] and CFT [24], have introduced adaptive weighting and long-range cross-modal interaction to enhance feature fusion. These studies confirm the importance of adaptive multispectral integration, but many of them also increase architectural complexity. This further motivates our choice of a lightweight scene-level gating design for real-time dual-stream RGBT detection.

Overall, existing adaptive fusion methods for RGBT detection can be broadly categorized into three groups: (1) channel-wise recalibration methods, which estimate modality importance through compact global descriptors; (2) spatial attention methods, which assign location-dependent fusion weights; and (3) transformer/cross-attention methods, which explicitly model long-range cross-modal interactions. Compared with the latter two categories, our GFM belongs to the first group and is intentionally designed as a lightweight scene-level gating unit. Its goal is not to maximize fusion granularity, but to provide low-cost adaptive modality calibration for real-time deployment.

### 2.2. Small Object Detection: The Downsampling Dilemma

Detecting small objects—such as distant pedestrians occupying fewer than 32 × 32 pixels—remains a critical challenge in thermal imagery due to the absence of rich texture and chromatic cues. The core issue fundamentally originates from the downsampling strategy embedded in modern convolutional backbones.

Modern one-stage detectors such as YOLOv8 [3], YOLOv9 [25], and YOLOv11 typically expand the receptive field by using stride = 2 convolutions. This design reduces computational cost, but it also decreases spatial resolution as the feature maps become progressively smaller. YOLOv9 improves gradient propagation through its Programmable Gradient Information mechanism; however, the spatial compression introduced during forward propagation remains unavoidable.

When small targets occupy only a limited number of pixels, repeated downsampling can easily weaken or even remove their structural responses. From a sampling viewpoint, once high-frequency details are compressed beyond a certain level, the lost information cannot be recovered in later stages. Sunkara et al. [12] observed that small thermal objects may disappear entirely in deeper layers when aggressive stride = 2 operations are applied.

One direct way to mitigate this issue is to add a P2 detection head (stride 4), as done in QueryDet [26]. The additional high-resolution branch improves recall for small objects, but it also increases memory usage and inference time. In practice, latency can rise by more than 30%, which limits its applicability on edge devices. Other approaches, such as MDCENet [27], attempt to compensate for small-target degradation through complex cross-dimensional feature enhancement. Although these designs enhance representation capability, they still operate on feature maps that have already undergone resolution reduction, and maintaining real-time performance remains challenging.

Rather than introducing extra prediction heads, our method revisits the downsampling operation itself. We adopt Space-to-Depth (SPD) convolution [12] to reorganize spatial information into the channel dimension. This transformation preserves local structural details while keeping the feature map size compact. As a result, fine-grained thermal cues can be retained in deeper layers without substantially increasing computational burden, which is more suitable for edge deployment scenarios.

### 2.3. Lightweight Architectures: The Dual-Stream Burden

Real-time RGBT detection introduces a distinct efficiency bottleneck: dual-stream backbones inherently double the parameter count and floating-point operations (FLOPs) compared to single-modal detectors.

Among modern one-stage detectors, the YOLO family has evolved from a fast general-purpose detection framework into a series of architectures offering increasingly favorable trade-offs between accuracy, speed, and deployment efficiency. In particular, lightweight and nano-scale variants are especially attractive for resource-constrained scenarios due to their compact parameter size, efficient inference characteristics, and mature engineering ecosystem. Although YOLO-based models are not inherently optimal for small-object detection, especially under multispectral and low-contrast conditions, they still provide a unified and deployment-friendly foundation for real-time detection. This is precisely why we build GEM-YOLO upon a lightweight YOLO-based framework: rather than replacing the detector family entirely, we aim to address its limitations in RGB-T fusion and small-object perception while preserving its practical efficiency advantages.

The YOLO family continues to dominate the efficiency–accuracy trade-off landscape. YOLOv8 [3] enhances gradient flow through the optimized C2f module. YOLOv10 [28] proposes NMS-free training based on consistent dual assignment to reduce post-processing latency. YOLOv11 further strengthens feature extraction capacity through refined C3k2 modules. However, directly extending these architectures to RGBT tasks substantially increases computational demands. For example, a naïve dual-stream YOLOv11n easily exceeds the FLOPs budget of many edge AI accelerators.

While recent lightweight models like FasterNet [29] and MobileNetV4 [30] explore highly efficient architectural paradigms, practical inference speed is not determined by FLOPs alone. As pointed out in [31] and further emphasized by the design guidelines of ShuffleNet V2 [32], hardware efficiency, memory access cost, and operator execution characteristics also play a critical role. Therefore, traditional lightweight operations (e.g., standard depthwise convolutions) may still suffer from low arithmetic intensity on GPUs, limiting their effectiveness in real-time GPU-oriented RGBT detection frameworks.

To mitigate this dual-stream burden, GEM-YOLO reconstructs the neck architecture using Ghost Modules [13] and GSConv [14]. Unlike YOLO-MS [33], which introduces complex multi-scale convolutional operations, Ghost modules reduce feature redundancy by generating intrinsic feature maps through inexpensive linear transformations. This lightweight reconstruction compensates for the additional overhead introduced by the fusion module and dual backbone. As a result, GEM-YOLO maintains total computational complexity below 9.0 GFLOPs while achieving superior efficiency–accuracy trade-offs compared with recent baselines such as RT-DETR [34].

## 3. The Proposed Method

In this section, we present the details of the proposed GEM-YOLO. The framework is designed to address the challenges of modal non-stationarity and small object feature loss in RGBT detection while maintaining real-time performance on edge devices. We first outline the overall architecture and then elaborate on the three core innovations: the Adaptive Multimodal Gated Fusion Mechanism (GFM), the Feature-Preserving Backbone via SPD-Conv, and the Lightweight Ghost-Neck.

### 3.1. Overall Architecture

**Figure 1:**
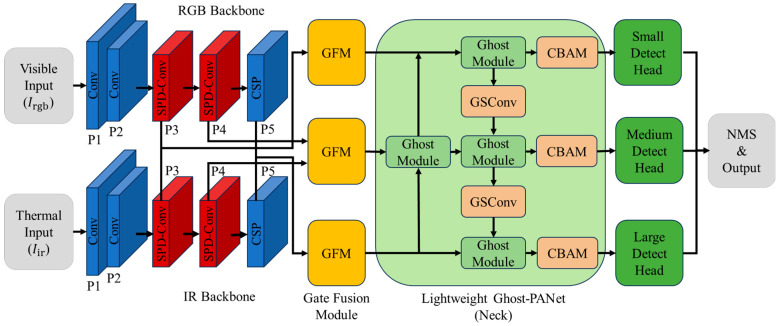
Overall architecture of GEM-YOLO. The network consists of a dual-stream RGB–IR backbone, multi-scale Gated Fusion Modules (GFM), and a lightweight Ghost-Neck integrated with CBAM. In the backbone, SPD-Conv is used to replace the conventional stride-2 downsampling convolutions at the corresponding stages, while the CSP feature extraction blocks are retained.

The overall architecture of GEM-YOLO is illustrated in Figure 1. Following the one-stage detection paradigm, the network comprises three logically distinct components optimized for efficiency and accuracy:

(a) Dual-Stream Decoupled Backbone: Two parallel CSP-Darknet backbones are employed to extract feature hierarchies from Visible ($I_{rgb}$) and Thermal ($I_{ir}$) images independently. To mitigate the loss of fine-grained spatial information during downsampling, we integrate Space-to-Depth (SPD) convolution modules at the low-resolution stages (specifically the P3 and P4 layers). Here, SPD-Conv is used to replace the original stride-2 downsampling convolutions at the corresponding backbone stages, rather than the CSP feature extraction blocks themselves. Separate backbone branches are intentionally preserved because visible and thermal modalities differ substantially in low-level contrast distributions, local structural patterns, and sensor-specific noise characteristics. While partial weight sharing is more efficient, it may suppress modality-specific representation learning at early stages. Therefore, GEM-YOLO prioritizes modality-decoupled feature extraction in the backbone, while the additional computational burden is alleviated later through the lightweight Ghost/GSConv neck.
(b) Multi-Scale Gated Fusion: Effective fusion requires the integration of features at multiple semantic levels [35]. We perform fusion at three distinct scales corresponding to downsampling strides of 8, 16, and 32. At each stage, the Gated Fusion Module (GFM) is deployed to dynamically calibrate the contribution of each modality based on global context, rather than simple linear superposition.
(c) Lightweight Semantic Aggregation: The fused features are aggregated using a Path Aggregation Network (PANet). To offset the computational overhead of the dual-stream backbone, the neck is reconstructed using Ghost Modules [13] and GSConv [14]. Furthermore, CBAM [36] attention blocks are embedded to refine the fused features before they are fed into the three decoupled detection heads.

It should be emphasized that the contribution of GEM-YOLO does not lie in proposing entirely new standalone low-level operators, but in the task-oriented integration of GFM, SPD-Conv, and the lightweight Ghost-Neck into a unified RGB-T detection framework for multispectral small-object perception and real-time deployment. More importantly, this design is not a straightforward stacking of existing modules. Instead, each component is introduced to address a specific limitation of dual-stream RGBT detection under edge constraints, namely scene-dependent modality calibration, preservation of fine-grained small-target structures during downsampling, and reduction in dual-stream computational overhead. Therefore, the overall architecture is problem-driven and complementary, rather than a generic aggregation of independent techniques.

### 3.2. Adaptive Multimodal Gated Fusion Mechanism (GFM)

In RGBT detection, the reliability of visible and thermal modalities is highly scene-dependent. Conventional fusion strategies, such as element-wise addition or channel concatenation, assign fixed importance to both modalities and therefore tend to propagate modality-specific noise into the fused representation. Recent attention-based fusion methods alleviate this issue by introducing channel-wise, spatial, or cross-modal attention. However, many of these mechanisms either rely on fine-grained spatial interaction or involve relatively heavy feature transformation, which increases computational overhead.

**Figure 2:**
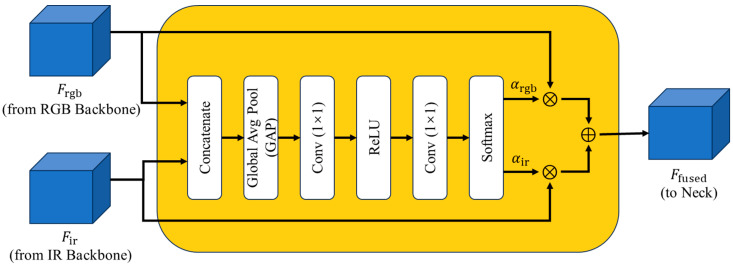
Structure of the Gated Fusion Module (GFM). Global Average Pooling captures scene context, and Softmax ensures adaptive weight allocation.

To achieve a better balance between adaptivity and efficiency, we propose a lightweight Gated Fusion Module (GFM) based on global-context gating, as shown in Figure 2. Unlike pixel-level spatial attention or transformer-style cross-attention, GFM does not model dense cross-location interactions. Instead, it summarizes the joint RGB–IR feature response through Global Average Pooling (GAP), and then predicts a pair of complementary modality confidence scores via a compact MLP followed by Softmax normalization. In this way, GFM performs scene-level modality calibration with minimal additional parameters and FLOPs, which is more suitable for real-time dual-stream RGBT detection.

Let $F_{rgb},F_{ir}\in {\mathbb{R}}^{C\times H\times W}$ denote the input feature maps. The fusion process is defined as follows:

1. Joint Feature Embedding:

First, the features are concatenated along the channel dimension. A Global Average Pooling (GAP) operation is then applied to compress the spatial information into a channel descriptor v, capturing the global environmental context:

(1) $$v=\mathrm{GAP}(\mathrm{Concat}(F_{rgb},F_{ir})),\;\;\;\;v\in {\mathbb{R}}^{2C\times 1\times 1}.$$

2. Modality Weight Learning:

A lightweight Multi-Layer Perceptron (MLP) is employed to model the non-linear relationship between modalities. The MLP consists of two 1 × 1 convolutional layers separated by a ReLU activation. Finally, a Softmax function generates the normalized complementary weights α:

(2) $$[\alpha_{rgb},\alpha_{ir}]=\mathrm{Softmax}(W_{2}\cdot \delta (W_{1}\cdot v)),$$

where δ denotes the ReLU function, and $W_{1},W_{2}$ are learnable weights. $\alpha_{rgb}$ and $\alpha_{ir}$ represent the global confidence scores for the RGB and IR streams, respectively.

3. Adaptive Fusion:

The final fused feature $F_{out}$ is obtained via a weighted summation:

(3) $$F_{out}=\alpha_{rgb}\cdot F_{rgb}+\alpha_{ir}\cdot F_{ir}.$$

This mechanism allows the network to adaptively suppress the noise from the ineffective modality based on global scene illumination and thermal conditions.

It should be noted that the proposed GFM performs scene-level modality calibration rather than spatially varying reliability estimation. Since the gating weights are generated from globally pooled RGB–IR descriptors, the same pair of modality weights is applied to all spatial locations within a feature map. This design is effective for low-cost global fusion under dominant scene conditions, such as low illumination or thermal degradation, but it may be less flexible in spatially heterogeneous scenes where modality reliability varies across different image regions. Nevertheless, this lightweight formulation is intentionally adopted to maintain negligible overhead and stable real-time performance in dual-stream edge deployment. Spatially adaptive gating will be explored in future work.

### 3.3. Feature-Preserving Backbone via High-Precision Light SPD-Conv

Detecting small objects (e.g., distant pedestrians) remains a persistent bottleneck in thermal imaging. Standard Convolutional Neural Networks (CNNs) commonly employ strided convolutions (e.g., stride = 2) for downsampling. This operation reduces the spatial resolution of feature maps and may weaken fine-grained local structures that are important for representing small targets. From a signal-preservation perspective, this effect can be understood as conceptually analogous to undersampling, where insufficient spatial resolution makes small-scale details harder to preserve. However, in CNNs, feature maps are discrete learned representations rather than continuously sampled signals. Therefore, this analogy is used only to provide intuition, rather than as a strict theoretical application of the Nyquist-Shannon sampling theorem. For small thermal targets, repeated downsampling may still lead to feature attenuation or even feature collapse in deeper layers.

**Figure 3:**
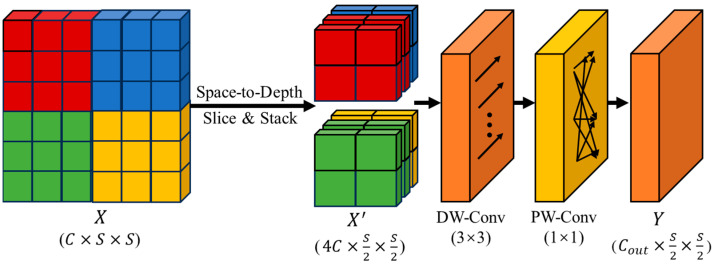
Diagram of the SPD-Conv. It combines lossless Space-to-Depth transformation with efficient Depthwise Separable Convolution.

To preserve fine-grained information while maintaining efficiency, we propose a High-Precision Lightweight SPD-Conv. As shown in Figure 3, this module integrates Space-to-Depth (SPD) transformation with Depthwise Separable Convolution. Given an input tensor $X\in {\mathbb{R}}^{C\times S\times S}$, the SPD layer first slices spatial blocks of size 2 × 2 and stacks them into the channel dimension, performing lossless downsampling:

(4) $$X^{\prime }=\mathrm{Space}2\mathrm{Depth}(X),\;\;\;\;X^{\prime }\in {\mathbb{R}}^{4C\times \frac{S}{2}\times \frac{S}{2}}.$$

To process the expanded channels efficiently, we apply a Depthwise Convolution (DW-Conv) with group size equal to channel number, followed by a Pointwise Convolution (PW-Conv) to project the channel dimension back to $C_{out}$:

(5) $$Y=\mathrm{SiLU}(\mathrm{BN}({\mathrm{PW}-\mathrm{Conv}}_{1\times 1}({\mathrm{DW}-\mathrm{Conv}}_{3\times 3}(X^{\prime })))).$$

For a standard convolution with a kernel size k×k, input channels $C_{in}$, output channels $C_{out}$, and output spatial size H×W, the computational cost is

(6) $${\mathrm{FLOPs}}_{std}=HWC_{in}C_{out}k^{2}.$$

In contrast, the depthwise separable convolution used after the SPD transform requires

(7) $${\mathrm{FLOPs}}_{ds}=HWC_{in}k^{2}+HWC_{in}C_{out}.$$

Therefore, the relative reduction depends on k and $C_{out}$. Under the common setting of k=3 and $C_{in}=C_{out}$, the cost ratio becomes

(8) $$\frac{{\mathrm{FLOPs}}_{ds}}{{\mathrm{FLOPs}}_{std}}=\frac{9+C_{out}}{9C_{out}}.$$

When $C_{out}$ is sufficiently large, this ratio approaches $\frac{1}{9}$, corresponding to an approximate reduction of 88.9%. For example, when $C_{out}=64$, the reduction is about 87.3%. Therefore, instead of claiming a fixed 87.5% reduction, we state that the proposed SPD-Conv block can substantially reduce convolutional computation relative to standard convolution, typically by about 87–89% under common settings. This reduction arises from replacing a standard convolution with a depthwise separable convolution after SPD rearrangement, rather than from the SPD operation itself.

### 3.4. Lightweight Ghost-Neck with Attention Refinement

Using a dual-stream backbone inevitably increases the number of parameters and computational cost, since features are extracted separately from two modalities. This additional overhead makes real-time deployment on edge hardware more demanding.

**Figure 4:**
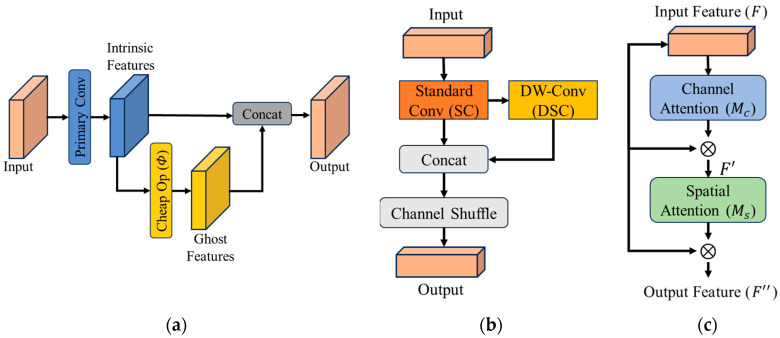
Illustration of the lightweight neck components: (**a**) Ghost Module, (**b**) GSConv, and (**c**) CBAM.

To address this issue, we redesign the neck with three efficiency-oriented modifications aimed at reducing redundant computation while preserving feature interaction. The overall structure of the redesigned neck is shown in Figure 4.

To reduce computational cost, the standard bottleneck blocks are replaced with Ghost Modules [13]. Based on the observation that feature maps exhibit high redundancy, the Ghost Module generates a few “intrinsic” maps using primary convolution and produces the rest via cheap linear operations (Φ), reducing FLOPs by approximately 50%:

(9) $$Y_{ghost}=\Phi (Y_{intrinsic}),$$

(10) $$Y_{out}=\mathrm{Concat}([Y_{intrinsic},Y_{ghost}]).$$

For the downsampling layers in the neck, we adopt GSConv [14] to balance efficiency and feature interaction. GSConv integrates standard convolution with depthwise separable convolution and introduces a channel shuffle operation to encourage cross-channel information exchange.

Compared with using depthwise convolution alone, this design maintains stronger feature connectivity while keeping computational cost close to that of lightweight separable operations:

(11) $$F_{GS}=\mathrm{Shuffle}(\mathrm{Concat}(F_{SC}(X),F_{DSC}(X))).$$

To further enhance feature representation, we incorporate the Convolutional Block Attention Module (CBAM) [36] into the neck. CBAM applies attention along the channel dimension and then refines the feature map spatially, allowing the network to emphasize more informative responses while suppressing less relevant ones.

(12) $$F^{\prime }=M_{c}(F)⊗F,$$

(13) $$F^{\prime \prime }=M_{s}(F^{\prime })⊗F^{\prime },$$

where $M_{c}$ and $M_{s}$ denote channel and spatial attention maps. This ensures the network focuses on discriminative regions in both thermal and visible domains.

## 4. Experiments

### 4.1. Dataset

The detailed statistics and data split settings of the two datasets are listed in Table 1.

#### 4.1.1. FLIR

The Teledyne FLIR dataset is a standard and highly challenging benchmark designed to encourage research in multi-sensor autonomous perception. It provides paired thermal infrared and visible-light images captured under various scenarios, including daytime, nighttime, and adverse weather conditions. The dataset primarily focuses on autonomous driving contexts, containing annotations for three main object categories: Person, Car, and Bicycle.

For our experiments, we utilized a refined set of 5136 image pairs with a resolution of 640 × 512, providing rich thermal radiation cues that are critical for detecting objects in visually degraded environments. Although the FLIR dataset provides an official train/test split, in this work, we re-partitioned the refined dataset into training, validation, and testing subsets using a unified 7:2:1 protocol in order to maintain a consistent evaluation setting with M3FD, to reserve an independent validation set for model selection and ablation studies, and to ensure that all compared methods were evaluated under exactly the same data split. The partition was generated once using a fixed random seed of 42 and was kept identical for all compared methods. Specifically, this split yielded 3595 pairs for training, 1027 pairs for validation, and 514 pairs for testing. This dataset serves as a robust testbed to evaluate the model’s feature-level fusion capabilities and overall detection accuracy.

#### 4.1.2. M3FD

The Multi-Modal Multispectral Fusion Dataset (M3FD) is a comprehensive dataset specifically constructed for visible-infrared image fusion and target detection. Unlike the FLIR dataset, M3FD provides perfectly aligned pairs of visible and infrared images, eliminating the interference of spatial offset and allowing the network to fully exploit pixel-to-pixel multimodal complementary information.

The dataset features a high resolution of 1024 × 768 and covers a wide range of complex illumination and environmental scenarios. It contains extensive bounding box annotations distributed across six target classes: Person, Car, Bus, Motorcycle, Truck, and Lamp. Consistent with our experimental protocol, a total of 4200 image pairs were divided into training, validation, and testing subsets at a 7:2:1 ratio. To ensure strict reproducibility and fair comparison, the partition was generated using the same fixed random seed of 42 and was shared by all competing methods. This resulted in 2940 pairs for training, 840 pairs for validation, and 420 pairs for testing. The strict alignment and diverse category distribution of M3FD make it an ideal dataset to validate the precision of our proposed Gated Fusion Module and the feature-preserving capabilities of the SPD-Conv structure for small target detection.

**Figure 5:**
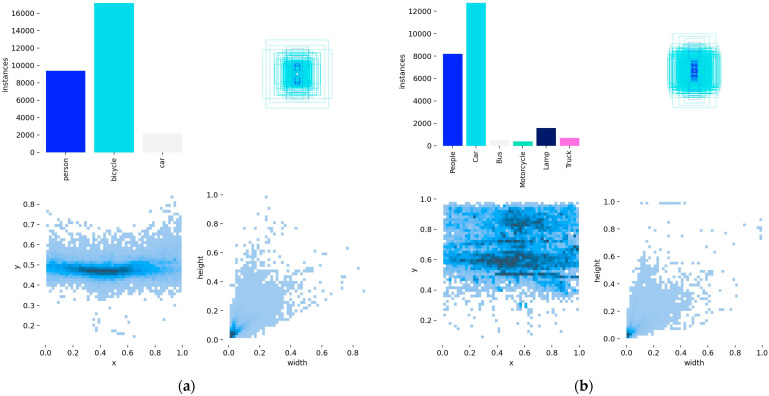
Statistical distributions of the experimental datasets, (**a**) FLIR and (**b**) M3FD. Each group of subplots presents category frequencies, bounding box layouts, spatial distributions of target centers, and width–height heatmaps. A considerable number of objects appear at small scales.

To better illustrate the dataset characteristics, Figure 5 presents the detailed label distributions for (a) FLIR and (b) M3FD. Both datasets exhibit natural class imbalances typical of real-world scenarios. Notably, the width-versus-height distribution heatmaps (bottom-right of each subfigure) reveal that target sizes are heavily concentrated near the origin. This overwhelming proportion of exceedingly small targets poses a severe risk of feature collapse during standard downsampling operations, which strongly justifies our integration of the High-Precision Lightweight SPD-Conv module to preserve fine-grained spatial information.

**Figure 6:**
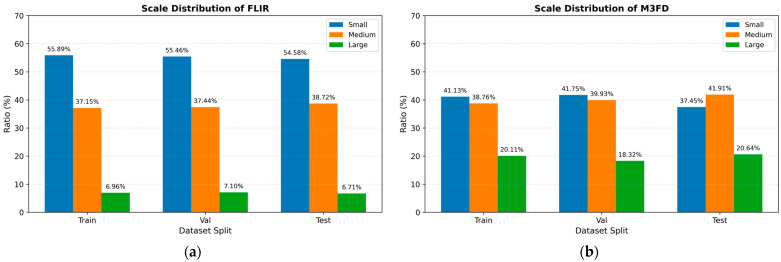
Scale distribution statistics of objects in the benchmark datasets according to the MS-COCO size protocol, where objects are divided into small $\left(area<32^{2}\right)$, medium $\left(32^{2}\leq area<96^{2}\right)$, and large $\left(area\geq 96^{2}\right)$ categories: (**a**) FLIR; (**b**) M3FD.

To better characterize the scale properties of the benchmark datasets, we further analyzed the object size distributions of FLIR and M3FD according to the MS-COCO size protocol, where objects were divided into small $\left(area<32^{2}\right)$, medium $\left(32^{2}\leq area<96^{2}\right)$, and large $\left(area\geq 96^{2}\right)$ groups based on their bounding-box area. The corresponding statistics are summarized in Table 2, while the scale distribution ratios are further visualized in Figure 6.

It can be observed that the two datasets exhibit different scale characteristics. The FLIR dataset is strongly dominated by small-scale targets, which account for more than 54% of the objects in all three subsets, while large targets constitute only about 7%. In contrast, the M3FD dataset presents a relatively more balanced distribution between small and medium objects. Specifically, small objects account for about 37–42%, medium objects account for about 39–42%, and large objects account for approximately 18–21%.

Overall, both datasets are dominated by small- and medium-scale targets, while large objects occupy a much smaller proportion. This distribution indicates that scale-sensitive evaluation is necessary for a rigorous assessment of multispectral detection performance, especially when verifying whether the proposed SPD-Conv module is particularly beneficial for small-object detection.

### 4.2. Experimental Environment and Setting

The details of the experimental hardware and software environments are summarized in Table 3. All experiments were conducted on a workstation equipped with a single NVIDIA GeForce RTX 4090 GPU (24 GB). The proposed network was implemented based on the Ultralytics YOLO framework using PyTorch.

The specific hyperparameter settings for the training phase are detailed in Table 4. The model was trained from scratch for 150 epochs with a batch size of 16. We unified the input image size to 640 × 640 using standard letterbox resizing. The network was optimized using Stochastic Gradient Descent (SGD) coupled with a cosine annealing learning rate scheduler. To ensure early training stability, a standard warmup strategy (typically 3 epochs) was employed. During training, the total loss was calculated as a weighted sum of the classification loss (BCE Loss), bounding box regression loss (CIoU Loss), and Distribution Focal Loss (DFL). Notably, to guarantee the convergence stability of the multi-modal feature fusion and prevent numerical underflow, Automatic Mixed Precision (AMP) was explicitly disabled.

For fairness, all compared models were trained under identical experimental settings, including the same data split, input resolution, optimizer configuration, and 150 training epochs unless otherwise specified.

To ensure strict reproducibility, the train/validation/test partition was generated once using a fixed random seed of 42 and then reused unchanged for all models and all repeated runs. After fixing the data split, all deep models were independently trained five times with different training seeds (42–46) to account for the statistical variability inherent in neural network optimization. The results reported in Table 5 and Table 7 are given as mean ± standard deviation over these five runs. In addition, statistical significance analysis was performed against the YOLOv11n baseline, and statistically significant improvements are marked with * (*p* < 0.05).

### 4.3. Experimental Evaluation Metrics

To comprehensively and objectively evaluate the performance of the proposed detection model, we employ several widely adopted evaluation metrics. These metrics assess both the detection accuracy and the computational efficiency of the network.

1. Accuracy Metrics (P, R, and mAP)

Detection accuracy is evaluated using Precision (P), Recall (R), and mean Average Precision (mAP). Precision measures the ratio of correctly predicted positive observations to the total predicted positives, while Recall calculates the ratio of correctly predicted positive observations to all observations in the actual class. They are defined as follows:

(14) $$P=\frac{TP}{TP+FP}\times 100\%$$

(15) $$R=\frac{TP}{TP+FN}\times 100\%$$

where TP, FP, and FN denote the number of True Positives, False Positives, and False Negatives, respectively. In the context of our multi-modal task, high Recall is particularly crucial to ensure that small or distant targets are not missed in visually degraded scenes.

The Average Precision (AP) for a single class is calculated as the area under the Precision-Recall (P-R) curve. The mAP is the average of the AP values across all N categories (where N=3 for FLIR and N=6 for M3FD):

(16) $$mAP=\frac{1}{N}\sum_{i=1}^{N}\int_{0}^{1}P(R)dR$$

Specifically, we report two standard mAP metrics:

- mAP@50 (%): The mean Average Precision calculated at a relatively loose Intersection over Union (IoU) threshold of 0.5. It reflects the model’s basic ability to locate and classify targets.
- mAP@50:95 (%): The average mAP calculated at different IoU thresholds ranging from 0.5 to 0.95 with a step size of 0.05. This is a much stricter metric that heavily penalizes imprecise bounding boxes, thereby effectively reflecting the model’s high-precision localization capability for small targets.

2. Efficiency Metrics (Params, GFLOPs, FPS, and Latency)

To validate the efficiency and lightweight design of our proposed architecture, we evaluate the model complexity and practical inference efficiency using the number of Parameters, Giga Floating-Point Operations, Frames Per Second (FPS), and Latency.

- Params (M): Measured in millions, this metric reflects the spatial complexity and memory footprint of the model.
- GFLOPs (G): Measured in billions of operations, this metric represents the time complexity and computational cost of the network. It should be emphasized that GFLOPs quantify theoretical computational complexity, but do not directly correspond to actual runtime latency, which is also influenced by hardware architecture, memory bandwidth, operator implementation, and parallel execution efficiency.
- FPS: This metric denotes the number of images processed per second during inference and is used to assess the real-time capability of the model.
- Latency (ms): This metric denotes the average inference time required to process a single image, directly reflecting the response speed of the model in practical deployment.

Reporting these four metrics together is important because Params and GFLOPs characterize the compactness and theoretical complexity of the model, while FPS and Latency provide direct evidence of its actual runtime efficiency. This comprehensive evaluation demonstrates that the integration of the Gated Fusion Module (GFM) and the lightweight SPD-Conv mechanism improves detection accuracy while preserving lightweight and real-time deployment capability.

## 5. Results

### 5.1. Comparative Experimental Results and Analysis

To thoroughly evaluate the performance of our proposed network, we conducted quantitative comparisons with several advanced YOLO-series object detectors on the FLIR dataset. Table 5 reports the mean ± standard deviation over five independent runs, including both overall metrics and class-wise results.

The comparison in this work mainly focuses on the YOLO-nano family, since GEM-YOLO is designed for RGB-T detection under lightweight and real-time deployment constraints. Nano-scale baselines, therefore, provide the most relevant reference in terms of parameter budget and computational complexity. In addition, several representative multimodal methods, including MBNet, ProbEn, and dual-stream RT-DETR, are also included to provide a broader comparison. Transformer-based RGBT methods such as CFT and GAFF have demonstrated promising detection performance. However, these methods usually involve more complex cross-modal interaction and attention operations, leading to a higher computational cost than the lightweight framework targeted in this work. Therefore, the experimental comparison in this paper mainly emphasizes compact and deployment-oriented baselines under resource-constrained settings. It should be noted that the efficiency comparison is made under the same RGB-T setting, and the reported GFLOPs reduction is calculated relative to the dual-stream YOLOv11n baseline.

Table 5 summarizes the quantitative results on the FLIR dataset. From the accuracy side, GEM-YOLO achieves the competitive overall Precision, mAP@50, and mAP@50:95, reaching 84.5 ± 0.2%, 82.8 ± 0.2%, and 46.5 ± 0.2%, respectively. Relative to the dual-stream YOLOv11n baseline, these values increase from 83.2 ± 0.2% to 84.5 ± 0.2% in Precision, from 81.8 ± 0.2% to 82.8 ± 0.2% in mAP@50, and from 45.6 ± 0.2% to 46.5 ± 0.2% in mAP@50:95. This suggests that the proposed design improves feature extraction and fusion while keeping localization performance stable.

At the class level, the proposed method gives the highest Precision in all three categories. For Person and Bicycle, it also achieves the highest mAP@50, reaching 83.8 ± 0.2% and 89.2 ± 0.2%, respectively. On the Car category, GEM-YOLO obtains 75.5 ± 0.3% mAP@50. Although this is slightly below a comparable result (75.8 ± 0.3%) achieved by dual-stream YOLOv8n, the gap is very small. Under the stricter mAP@50:95 criterion, the proposed method remains competitive across categories, and its Bicycle result reaches 58.3 ± 0.2%, indicating stable performance on small and structurally complex targets.

It is worth noting that GEM-YOLO does not uniformly outperform all competing methods across every category. In particular, on the Car category, its result is slightly lower than that of dual-stream YOLOv8n by 0.3 percentage points. We believe this is mainly because the proposed design is more strongly optimized for small and medium targets, where preserving fine-grained structures during downsampling is especially important. By contrast, large vehicle targets are less sensitive to spatial detail loss caused by conventional stride-based downsampling, so the relative advantage of SPD-Conv becomes less pronounced. In addition, the lightweight neck reconstruction in GEM-YOLO is designed to improve efficiency under dual-stream constraints, which may slightly reduce the feature redundancy beneficial to large-object representation. Therefore, while GEM-YOLO maintains competitive performance on the Car category, its gain is more evident on smaller and structurally more challenging targets.

Besides the accuracy improvement, GEM-YOLO also keeps the model lightweight. Its parameter count is 3.44 M and its computational cost is 7.58 GFLOPs, both lower than those of the dual-stream YOLOv11n baseline (3.79 M and 9.31 GFLOPs). This corresponds to an 18.6% reduction in GFLOPs relative to the same dual-stream baseline. Compared with MBNet, ProbEn, and dual-stream RT-DETR, the proposed method also uses a clearly smaller model scale.

A similar pattern can be observed in runtime performance. GEM-YOLO runs at 476.2 ± 3.5 FPS with a latency of 2.1 ± 0.02 ms, outperforming MBNet (285.7 ± 2.5 FPS, 3.5 ± 0.03 ms), ProbEn (263.2 ± 2.2 FPS, 3.8 ± 0.03 ms), and dual-stream RT-DETR (70.4 ± 0.9 FPS, 14.2 ± 0.15 ms). Although dual-stream YOLOv10n reaches a higher FPS (666.7 ± 5.3), its detection accuracy is weaker, especially under mAP@50:95. This means that the proposed method reduces the extra cost introduced by RGB-T dual-stream processing without giving up practical real-time capability.

In general, the results in Table 5 show that GEM-YOLO offers a better balance between speed and accuracy on the FLIR dataset. The small standard deviations over five independent runs also indicate that the performance remains stable across repeated experiments. For resource-limited multispectral detection tasks, this balance is important in practice.

The original YOLOv11n requires 4.67 GFLOPs, but it is limited to single-modal input, as shown in Table 6. For RGB-T detection, the network needs to process visible and infrared data simultaneously, which increases the computational cost of the dual-stream YOLOv11n baseline to 9.31 GFLOPs. With the lightweight design adopted in the proposed method, this value is reduced to 7.58 GFLOPs, corresponding to an 18.6% reduction relative to the dual-stream baseline.

**Figure 7:**
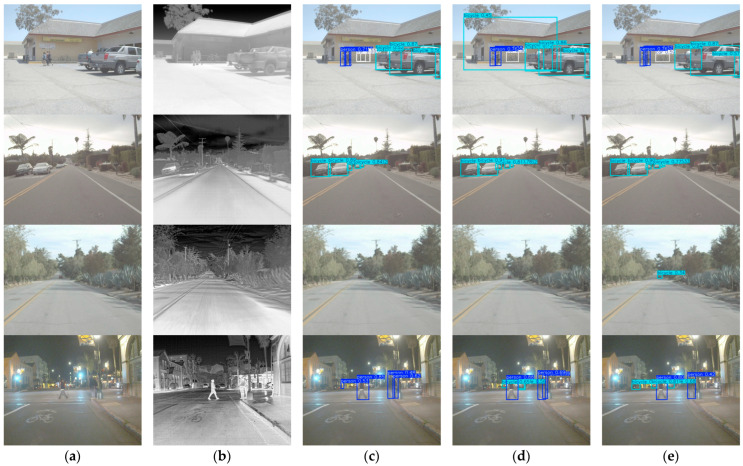
Visual comparison of different methods on the FLIR dataset. Columns (**a**–**e**) show the RGB image, infrared image, MBNet result, Dual-stream YOLOv11n result, and the result of the proposed method, respectively. Each row corresponds to a representative scene.

Figure 7 shows qualitative comparisons on the FLIR dataset in four representative scenes. The RGB images, infrared images, and detection results are displayed side by side for direct visual inspection.

In the first three rows, the proposed method gives more complete detections overall. In the first row, all methods detect the main targets, but the proposed method produces more stable predictions. In the second row, where the targets are small and far from the camera, the proposed method still maintains valid detections with relatively higher confidence scores, while MBNet and Dual-stream YOLOv11n show weaker responses and partial missed detections. The third row is more challenging, and only the proposed method gives a valid prediction, whereas both MBNet and Dual-stream YOLOv11n fail to detect the target.

The fourth row shows a more difficult nighttime case, in which the advantage of GEM-YOLO becomes less pronounced. In this scene, the RGB modality is weakened by low illumination, while the infrared modality is also disturbed by background thermal interference, so both modalities provide less reliable cues for fusion. As a result, the confidence scores of the proposed method are not consistently superior, and the detection result does not clearly outperform the strongest neighboring baseline. This case indicates that the current global gating strategy still has limitations when both modalities are simultaneously degraded.

Overall, the proposed method shows fewer missed detections and more reliable confidence scores in most cases, especially for small or distant targets, while the fourth-row example also suggests that further improvement is still needed in harder nighttime scenes.

**Figure 8:**
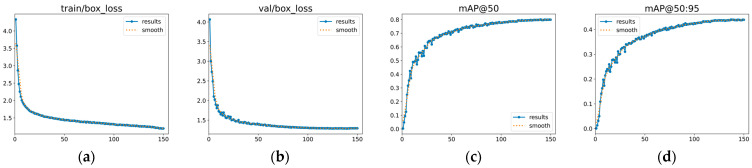
Training and validation curves of GEM-YOLO on the FLIR under the 150-epoch training schedule. (**a**) Training box loss. (**b**) Validation box loss. (**c**) Validation mAP@50. (**d**) Validation mAP@50:95.

The training curves of the proposed GEM-YOLO on the FLIR dataset are presented in Figure 8. Both the training and validation box losses decrease steadily and gradually become stable as the number of epochs increases. Meanwhile, the validation mAP@50 and mAP@50:95 continue to improve before leveling off in the later stage of training. Overall, these trends indicate that the model converges normally under the 150-epoch setting, without showing obvious signs of severe overfitting.

To further validate the generalization and robustness of the proposed method in diverse scene conditions, experiments were also conducted on the well-aligned M3FD dataset. The quantitative results over six categories are reported in Table 7 as mean ± standard deviation over five independent runs.

The results in Table 7 indicate that GEM-YOLO performs well on the M3FD dataset while keeping the model lightweight. With only 3.44 M parameters and 7.58 GFLOPs, it is smaller than the dual-stream YOLOv11n baseline (3.79 M parameters and 9.31 GFLOPs). This means that the computational cost is reduced by 18.6% under the same dual-stream RGB-T setting. Compared with MBNet, ProbEn, and dual-stream RT-DETR, the proposed method also keeps a clear advantage in model size.

From the detection results, GEM-YOLO reaches 69.0 ± 0.3% mAP@50 and 43.8 ± 0.3% mAP@50:95. Relative to the dual-stream YOLOv11n baseline, the improvement is 2.5 points in mAP@50 and 1.2 points in mAP@50:95. This shows that the proposed design brings gains in both recognition and localization, rather than only reducing computation.

The category-level results further reflect this trend. For Person and Truck, the proposed method achieves the highest mAP@50, reaching 78.8 ± 0.3% and 72.1 ± 0.4%, respectively. The result on Bus is also the best in the table, with 80.2 ± 0.4% mAP@50. Under the stricter mAP@50:95 metric, GEM-YOLO still ranks first on Person, Bus, and Truck, with 46.0 ± 0.3%, 60.5 ± 0.3%, and 47.2 ± 0.4%. These results suggest that the method remains stable on several object categories, especially when more precise localization is required.

In terms of speed, GEM-YOLO runs at 384.6 ± 3.2 FPS with a latency of 2.6 ± 0.02 ms. This is clearly faster than MBNet (285.7 ± 2.7 FPS, 3.5 ± 0.03 ms), ProbEn (263.2 ± 2.4 FPS, 3.8 ± 0.03 ms), and dual-stream RT-DETR (69.9 ± 1.0 FPS, 14.3 ± 0.15 ms). Although dual-stream YOLOv10n reaches a higher FPS (454.5 ± 3.8), its overall detection accuracy is lower. In other words, the proposed method keeps real-time capability without giving up too much accuracy.

Taken together, these results show that GEM-YOLO offers a practical balance between efficiency and detection performance on M3FD. In addition, the small standard deviations over five independent runs suggest that the method remains stable across repeated experiments.

**Figure 9:**
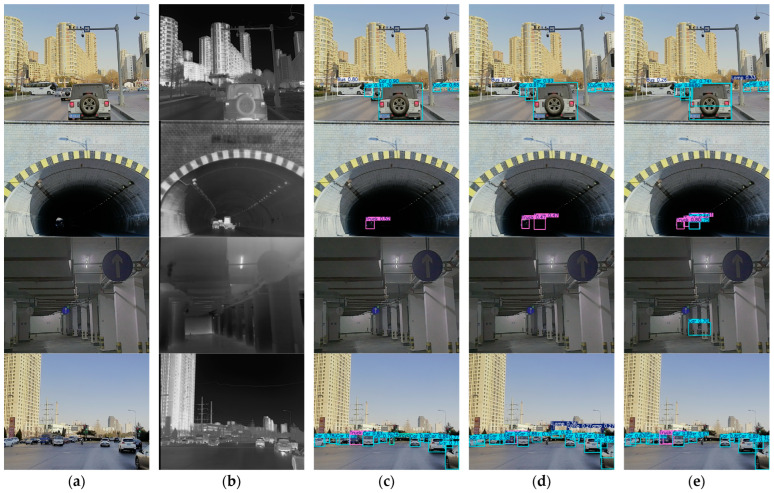
Visual comparison of different methods on the M3FD dataset. Columns (**a**–**e**) show the RGB image, infrared image, MBNet result, Dual-stream YOLOv11n result, and the result of the proposed method, respectively. Each row corresponds to a representative scene.

Qualitative comparisons on the M3FD dataset are presented in Figure 9 for four representative scenes. The RGB images, infrared images, and detection results are displayed side by side for direct visual comparison.

In the first three rows, the proposed method gives more complete detection results overall. In the first row, all methods detect the main vehicles, but the proposed method shows more stable predictions. In the second row, the target is small and appears in a low-contrast tunnel scene. Compared with MBNet and Dual-stream YOLOv11n, the proposed method gives a clearer response. In the third row, the target is located in a dim indoor scene, where the proposed method still maintains a valid detection, indicating better robustness under challenging illumination conditions.

The fourth row presents a more crowded urban scene. Although most major objects are detected, partial missed detection still appears. In particular, the lamp target is missed, which shows that the proposed method remains less effective for small or weak targets in cluttered scenes.

Overall, the proposed method achieves more reliable visual results in most cases. At the same time, the missed target in the fourth row suggests that there is still room for improvement in dense scenes and for small-category objects.

These results indicate that GEM-YOLO achieves a more favorable accuracy–efficiency trade-off than recent lightweight baselines, and even surpasses dual-stream YOLOv11n in mAP@50 on both FLIR and M3FD with substantially lower GFLOPs.

**Figure 10:**
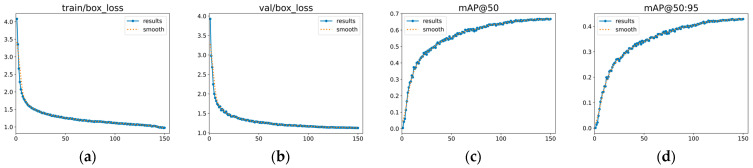
Training and validation curves of GEM-YOLO on the M3FD under the 150-epoch training schedule. (**a**) Training box loss. (**b**) Validation box loss. (**c**) Validation mAP@50. (**d**) Validation mAP@50:95.

As shown in Figure 10, the proposed GEM-YOLO exhibits stable training behavior on the M3FD dataset. Both the training and validation box losses decrease steadily and gradually level off as training proceeds. Meanwhile, the validation mAP@50 and mAP@50:95 continue to improve before becoming stable in the later stage. These curves suggest that the model converges normally under the 150-epoch setting, without obvious signs of severe overfitting on the M3FD dataset.

To further verify the practical deployment capability of the proposed model, we benchmarked representative methods on a Jetson Nano edge device, as shown in Table 8. Unlike Params and GFLOPs, which reflect theoretical model complexity, Latency and FPS directly indicate actual runtime efficiency and are influenced by hardware architecture, memory access, and parallel execution efficiency.

The proposed model achieves 26.0 FPS with a latency of 38.5 ms, outperforming all other deployable methods in edge-side inference speed. By comparison, Dual-stream YOLOv11n reaches 23.0 FPS, Dual-stream YOLOvXn reaches 22.7 FPS, and MBNet and ProbEn achieve only 14.0 FPS and 13.0 FPS, respectively. Dual-stream RT-DETR could not be stably benchmarked on Jetson Nano under the current configuration. These results demonstrate that the proposed lightweight design not only reduces theoretical complexity but also yields clear runtime advantages on resource-constrained platforms.

### 5.2. Ablation Study

**Figure 11:**
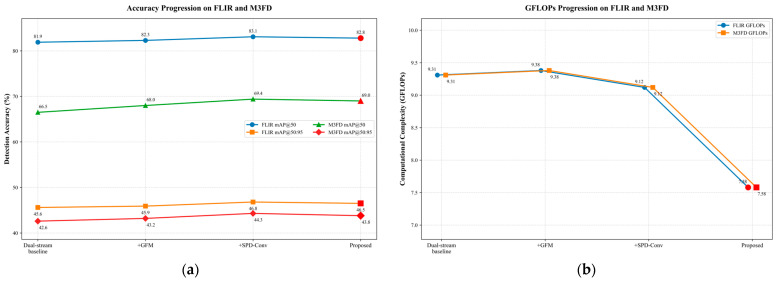
Ablation results of the proposed modules on the FLIR and M3FD datasets. (**a**) Changes in mAP@50 and mAP@50:95 across different ablation variants. (**b**) Changes in GFLOPs across the corresponding ablation variants.

To systematically validate the individual contributions of the proposed components in GEM-YOLO, comprehensive ablation experiments were conducted on both the FLIR and M3FD datasets. The baseline model is constructed using the state-of-the-art YOLOv11n architecture, extended into a dual-stream network with a naive channel concatenation fusion strategy. We incrementally integrated the Gated Fusion Module (GFM), Space-to-Depth Convolution (SPD-Conv), and the lightweight Ghost-Neck. The quantitative results and progressive performance variations are summarized in Table 9, and the corresponding trends are illustrated in Figure 11.

First, we integrated the GFM to replace the naive concatenation in the dual-stream baseline. As shown in Figure 11 and Table 9, this addition yields clear improvements in detection accuracy, particularly on the rigorously aligned M3FD dataset, where mAP@50 increases from 66.5% to 68.0%. Because the GFM utilizes a lightweight global gating mechanism incorporating Global Average Pooling and a compact MLP to dynamically calibrate modality weights, it introduces only negligible computational overhead (GFLOPs increase marginally from 9.31 to 9.38, and Params from 3.79 M to 3.82 M). This indicates that GFM is effective in suppressing modality-specific noise and enhancing complementary multispectral features under complex illumination conditions.

Building upon GFM, we then targeted the feature loss of small objects by introducing the SPD-Conv blocks. It should be noted that this step is a lightweight replacement rather than a naive addition. Specifically, we replaced the standard stride = 2 downsampling convolutions in the backbone with Space-to-Depth transformations followed by highly efficient depthwise separable convolutions. As shown in Table 9 and Figure 11, this replacement reduces the overall computational cost (GFLOPs drop to 9.12) while further improving detection accuracy to its best level among the intermediate ablation variants, with FLIR mAP@50 reaching 83.1% and M3FD reaching 69.4%. By shifting fine-grained spatial information into the channel dimension, SPD-Conv helps alleviate feature collapse and improves the localization of distant and small targets.

Finally, to further satisfy the efficiency requirements of real-time edge deployment, we reconstructed the feature aggregation neck using Ghost modules and GSConv. As shown in Figure 11 and the final row of Table 9, this lightweight design further reduces the computational complexity to 7.58 GFLOPs and the parameter count to 3.44 M. Although this compact neck design causes a slight fluctuation in absolute accuracy (e.g., FLIR mAP@50 changes from 83.1% to 82.8%), the final proposed model still maintains competitive detection performance on both datasets. Overall, the complete GEM-YOLO architecture achieves a favorable balance between multimodal detection accuracy and computational efficiency, which is important for real-time deployment on resource-constrained platforms.

To isolate the effect of the proposed fusion module, we compared the dual-stream baseline with naive concatenation against the same architecture equipped only with GFM. As shown in Table 10, replacing static concatenation with GFM improves FLIR mAP@50 from 82.2% to 82.5% and mAP@50:95 from 45.9% to 46.1%. On the more strictly aligned M3FD dataset, the gain is more pronounced, with mAP@50 increasing from 66.8% to 68.2% and mAP@50:95 from 42.9% to 43.4%, while the computational overhead remains marginal (only +0.03 M parameters and +0.07 GFLOPs). These results verify that the proposed GFM alone contributes consistent accuracy improvements through adaptive modality calibration.

As shown in Table 11, introducing the proposed SPD-Conv module consistently improves detection performance across different object scales on both datasets. More importantly, the most notable gains are observed on small objects. Specifically, on the FLIR dataset, the inclusion of SPD-Conv improves AP_s from 31.8 to 34.2, yielding a gain of +2.4. On the M3FD dataset, AP_s increases from 16.3 to 18.5, corresponding to a gain of +2.2. These results provide direct evidence that SPD-Conv is particularly beneficial for small-object detection, which is consistent with its design motivation of preserving fine-grained spatial details.

Meanwhile, the proposed module also brings stable improvements on medium and large objects. On FLIR, AP_m/AP_l increase by +0.6/+0.5, while on M3FD the corresponding gains are +0.7/+0.5. Although these improvements are relatively smaller than those on AP_s, they indicate that the enhancement in small-object detection is not achieved at the expense of degraded performance on other scales. Overall, the scale-wise ablation results demonstrate that the proposed SPD-Conv module improves detection performance in a consistent manner, with the most significant contribution concentrated on small-scale targets.

### 5.3. Scale-Wise Detection Analysis

The results in Table 12 provide clearer evidence of the small-object advantage of the proposed method. On both datasets, the highest AP_s (average precision for small objects) is obtained by the proposed model, reaching 34.2 on FLIR and 18.5 on M3FD. This suggests that the proposed design is particularly effective for detecting small targets. At the same time, its performance on medium and large objects remains competitive, and in some cases even stronger than the compared methods, with AP_m/AP_l values of 56.5/61.2 on FLIR and 54.8/73.9 on M3FD. Taken together, these results indicate that the improvement on small objects is not achieved at the expense of other object scales, which further supports the effectiveness of the proposed method under the MS-COCO size protocol.

### 5.4. Discussion

#### 5.4.1. Global Gating Behavior and Its Limitation

**Figure 12:**
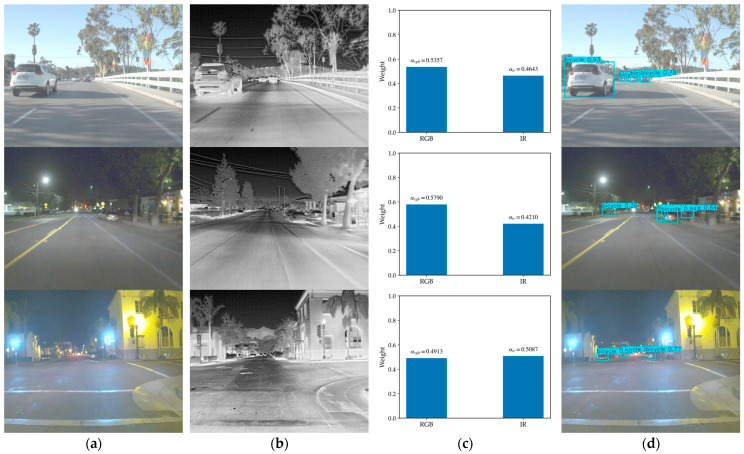
Visualization of the behavior of the proposed GFM under three representative scene conditions. The four columns correspond to (**a**) RGB image, (**b**) thermal infrared image, (**c**) predicted global modality weights, and (**d**) final detection result, respectively. The three rows represent a daytime scene, a nighttime scene, and a spatially heterogeneous scene, respectively.

The behavior shown in Figure 12 is in line with previous multispectral detection studies, which suggest that adaptive fusion is usually more effective than static fusion when the relative reliability of visible and thermal information changes across scenes [22,23,24]. As illustrated in Figure 12, the proposed GFM responds differently under three representative conditions. In the daytime case shown in the first row, the RGB branch is assigned a relatively larger weight, indicating that the detector tends to rely more on visible structural information when illumination is adequate. In contrast, for the nighttime example in the second row, the infrared branch becomes more dominant, which reflects the stronger stability of thermal cues under low-light conditions.

A different situation appears in the third row, which corresponds to a spatially heterogeneous scene containing local glare, dark areas, and uneven illumination within the same frame. In this case, the reliability of the two modalities is no longer consistent over the whole image. Even so, the predicted weights in Figure 12c show that the current GFM is still able to provide a reasonable scene-level fusion preference with only a very small computational overhead. This indicates that global modality calibration can capture the dominant cross-modal tendency of a scene, which is useful for real-time RGBT detection in resource-constrained settings.

At the same time, Figure 12 also reveals the limitation of the present design. Because the modality coefficients are derived from globally pooled features, the network assigns only one shared pair of weights to the entire frame. Consequently, the current GFM cannot explicitly describe local differences in cross-modal reliability, for example, when one region is more suitably represented by RGB information while another region is better characterized by thermal cues. This limitation also helps explain why the improvement brought by GEM-YOLO becomes less obvious in spatially heterogeneous scenes, where a single global weighting strategy may be insufficient to reflect conflicting local modality preferences. Therefore, the proposed fusion module is more appropriately understood as a lightweight scene-level calibration mechanism rather than a fully spatially adaptive interaction module. In essence, this is a deliberate trade-off between finer fusion granularity and deployment efficiency. Exploring lightweight spatially adaptive gating strategies would therefore be a meaningful direction for further improving local cross-modal calibration without causing a substantial increase in computational cost.

#### 5.4.2. Failure Cases and Balanced Error Analysis

Although GEM-YOLO achieves a favorable balance between detection accuracy and computational efficiency on both FLIR and M3FD, its advantages are not equally evident across all categories and scene conditions. The main limitations are mainly reflected in two situations: scenes where both modalities are degraded at the same time, and categories dominated by relatively large objects.

A difficult case arises when visible and thermal cues become unreliable simultaneously. In low-illumination scenes, the RGB branch may lose useful structural information because of weak contrast, while the thermal branch may also become less discriminative when the target is embedded in a warm background or affected by thermal crossover. Under such conditions, both modalities provide weak or ambiguous evidence. Similar phenomena have also been reported in previous multispectral studies, where the benefit of fusion becomes limited once both modalities degrade at the same time [22,23,24]. As a lightweight global scene-level gating module, the proposed GFM can still adjust the relative contributions of the two branches, but it cannot recover discriminative information that has already been severely weakened. As a result, the fused representation may remain insufficiently distinctive, leading to lower confidence or missed detections. This limitation is more noticeable in some challenging nighttime scenes, where the gain of GEM-YOLO becomes less obvious.

Another limitation appears in categories dominated by relatively large targets, especially the Car category on FLIR. As shown in Table 5, GEM-YOLO remains competitive on this category, but its mAP@50 is slightly lower than that of a neighboring dual-stream baseline. A possible explanation is that the main architectural benefits of GEM-YOLO are more relevant to small and medium targets. Previous studies have shown that repeated stride-based downsampling can easily weaken fine-grained target cues, especially for small, distant, or structurally weak objects [12,26,27]. In GEM-YOLO, SPD-Conv is introduced to alleviate this problem by preserving more local detail during downsampling. However, large vehicle targets are naturally less sensitive to such detail loss, since their spatial extent and semantic structure remain relatively stable even under conventional downsampling. Therefore, the gain brought by SPD-based feature preservation becomes less evident for large objects.

The lightweight neck reconstruction follows the same efficiency-oriented design logic. It helps reduce the computational cost introduced by dual-stream processing, which is beneficial for real-time deployment, but it also removes part of the feature redundancy retained in heavier architectures. For large-object categories, detection often depends more on high-level semantic completeness than on local fine-scale preservation. In such cases, an efficiency-oriented design may not always produce the strongest category-specific improvement. This trade-off helps explain why GEM-YOLO shows clearer gains on smaller and structurally more challenging targets, while remaining competitive rather than consistently superior in some large-object cases.

Overall, the results suggest that GEM-YOLO is more advantageous in scenarios that require a practical balance between efficiency and robust multispectral perception, particularly for small and medium targets in complex environments. At the same time, the current global gating strategy is still less flexible when modality reliability changes sharply across regions or when both modalities degrade simultaneously. Further improvement may therefore depend on more spatially adaptive fusion mechanisms and stronger cross-modal compensation strategies for these difficult conditions.

#### 5.4.3. Separate Versus Shared Backbone Design

Another important issue in dual-stream RGBT detection is whether the RGB and thermal branches should remain separate or share part of their parameters. Partial sharing, especially in shallow layers, is often viewed as an efficient option because it can reduce model size and computational cost, and may also help alleviate overfitting in some settings [5,6,7,20]. However, this choice also relies on an implicit assumption, namely that the low-level features of visible and thermal images can be described by a sufficiently common set of filters.

In the proposed framework, the two modalities still use separate backbone branches. The main reason is that RGB and thermal imagery differ markedly in their low-level characteristics, such as contrast distribution, texture statistics, and sensor-specific noise patterns [8,34]. Since early convolutional layers mainly encode local structures, gradients, and edges, introducing parameter sharing too early may weaken modality-specific information before adaptive fusion is carried out. This issue is particularly relevant in multispectral detection, because the visible branch is more sensitive to illumination and appearance details, whereas the thermal branch is more closely related to heat saliency and is influenced by different background interference mechanisms [22,23,24].

Under this design, preserving modality-specific backbone extraction is more consistent with the role of GFM. The proposed fusion module is applied after each branch has already formed its own feature representation, and its purpose is to adaptively rebalance the contributions of the two modalities. For this reason, the present framework does not reduce complexity by sharing early backbone parameters. Instead, the extra cost brought by the dual-stream structure is controlled mainly in the feature aggregation stage. In particular, the Ghost/GSConv-based neck helps remove redundancy while maintaining effective feature interaction, which is also consistent with existing studies on efficient network design [13,14,32]. In essence, this reflects a practical trade-off: the backbone preserves modality-specific representation capability, while efficiency is improved in later stages.

Even so, partial sharing remains a meaningful alternative for lightweight multimodal detection and may provide a different balance between accuracy and efficiency. A systematic comparison between separate and partially shared backbone strategies is beyond the scope of the current revision, but it is still worth further study. Future work will therefore examine whether carefully designed partial sharing can further improve efficiency without noticeably weakening the modality-specific representation capability required for robust RGBT detection.

#### 5.4.4. Discussion on Comparison Scope

In this work, the experimental comparison is mainly conducted with nano-scale detectors. This choice is closely related to the design objective of GEM-YOLO, which focuses on improving RGBT detection performance while maintaining a lightweight structure suitable for real-time deployment. Under this setting, nano-scale backbones provide a more appropriate reference, as they are better aligned with the target range of model size, computational cost, and practical edge deployment requirements [2,28,37].

By contrast, larger models such as YOLOv_s variants usually involve increased parameter scale and higher computational burden. Although such models may offer stronger feature representation in some cases, they also shift the comparison to a different accuracy–efficiency regime. Similar observations have been discussed in previous studies, where the performance of detection models is closely tied to the trade-off between representational capacity and computational efficiency [29,32]. Therefore, direct comparison with larger models is less consistent with the primary goal of this work.

For this reason, the current experiments focus on lightweight nano-scale baselines, which better reflect the intended application scenario of GEM-YOLO. A broader comparison across different model scales, including larger detectors, remains meaningful, but is more suitable as an extension of the present study rather than its main focus.

## 6. Conclusions

In this paper, we proposed GEM-YOLO, a real-time and lightweight RGBT object detector for improving the accuracy–efficiency trade-off of dual-stream multispectral detection on resource-constrained edge platforms. To address the limitations of insufficiently adaptive multimodal fusion, the loss of small-object features during downsampling, and the computational burden of dual-stream processing, we introduced three key architectural innovations. First, the Adaptive Multimodal Gated Fusion Mechanism (GFM) was designed to dynamically calibrate modality weights based on global context, thereby reducing modality-specific noise in complex environments. Second, Space-to-Depth (SPD) convolutions were integrated into the backbone to better preserve small-target information during downsampling. Finally, to improve runtime efficiency, the feature aggregation neck was reconstructed using Ghost modules and GSConv to reduce computational redundancy and improve runtime efficiency.

Extensive evaluations on the challenging FLIR and well-aligned M3FD datasets verify the effectiveness of the proposed method. Quantitative results demonstrate that GEM-YOLO provides a favorable balance between multispectral detection accuracy and computational cost. Operating at merely 7.58 GFLOPs with a compact size of 3.44 M parameters, the proposed network reduces the computational cost by 18.6% relative to the dual-stream YOLOv11n baseline. Concurrently, it achieves highly competitive mAP@50 scores of 82.8% on FLIR and 69.0% on M3FD, while exhibiting robust bounding box localization capabilities under the highly stringent mAP@50:95 metric. In addition, GEM-YOLO achieves competitive detection accuracy on both datasets while offering a more favorable accuracy–efficiency trade-off, and also demonstrates a competitive balance between detection performance and computational cost among the compared lightweight nano-scale detectors. Ultimately, GEM-YOLO provides a highly effective, robust, practical and promising solution for real-time all-weather object detection on resource-constrained edge platforms.

While GEM-YOLO is currently designed for the RGBT pair, the architecture is modality-agnostic and can potentially be extended to other sensor combinations such as LIDAR + camera, RGB-D, or multi-camera systems. Future work will focus on deployment-oriented validation on real portable edge devices and further evaluation under real-world multispectral sensing conditions. In addition, more fine-grained adaptive fusion strategies and broader comparisons with other lightweight detector families will be explored to further improve the robustness and generalization ability of the proposed framework. We also plan to release the trained model and code in a public repository to facilitate reproducibility and promote further research in lightweight multimodal detection.

## Figures and Tables

**Table 1:** Detailed statistics of FLIR and M3FD datasets. Both datasets are randomly split into training, validation, and testing sets with a ratio of 7:2:1.

Dataset	Total Pairs	Classes (Count)	Original Image Size	Input Image Size	Data Split (Train: Validation: Test)
FLIR	5136	3	640 × 512 (pixel)	640 × 640 (pixel)	7:2:1
M3FD	4200	6	1024 × 768 (pixel)		

**Table 2:** Object scale distributions of the FLIR and M3FD datasets based on the MS-COCO size protocol.

Datasets	Split	Images	Objects	Small	Medium	Large	Small Ratio	Medium Ratio	Large Ratio
FLIR	Train	3595	28,631	16,002	10,637	1992	55.89%	37.15%	6.96%
	Val	1027	7960	4415	2980	565	55.46%	37.44%	7.10%
	Test	514	4130	2254	1599	277	54.58%	38.72%	6.71%
M3FD	Train	2940	23,990	9867	9298	4825	41.13%	38.76%	20.11%
	Val	840	7080	2956	2827	1297	41.75%	39.93%	18.32%
	Test	420	3338	1250	1399	689	37.45%	41.91%	20.64%

**Table 3:** Experimental Environment.

	Environment
Hardware	Intel(R) Xeon(R) Gold 6430
	RAM 120 GB
	NVIDIA GeForce RTX 4090 24 GB × 1
Software	Windows 11
	Python 3.12.3
	PyTorch 2.2.2
	Cuda 11.8
	Ultralytics YOLO (v8.3.75)

**Table 4:** Experimental Setting.

Parameter	Value
Epochs	150
Batch size	16
Input size	640 × 640
Optimizer	SGD (lr0 = 0.01, momentum = 0.937, weight_decay = 0.0005)
Learning rate	Initial lr0 = 0.01; cosine decay to lr_final = 1 × 10^−4^ (lrf = 0.01)
Workers	8
Data Augmentation	Mosaic, HSV, Flip (Mosaic disabled in the last 10 epochs)
Precision (AMP)	False

**Table 5:** Quantitative comparison of detection accuracy and efficiency among state-of-the-art methods and our proposed model on the FLIR dataset.

Method	Classes	Precision (%)	Recall (%)	mAP@50 (%)	mAP@50:95 (%)	Params (M)	GFLOPs (G)	FPS	Latency (ms)
Dual-stream YOLOv5n	All	84.4 ± 0.3	73.2 ± 0.3	82.4 ± 0.3	45.7 ± 0.3	3.67	9.81	400.0 ± 3.2	2.5 ± 0.02
	Person	85.2 ± 0.3	74.1 ± 0.3	83.1 ± 0.3	44.3 ± 0.3				
	Bicycle	88.1 ± 0.3	80.0 ± 0.3	89.1 ± 0.3	57.9 ± 0.3				
	Car	80.0 ± 0.3	65.4 ± 0.4	74.9 ± 0.4	35.0 ± 0.4				
Dual-stream YOLOv8n	All	85.3 ± 0.2	73.0 ± 0.2	82.7 ± 0.2	46.5 ± 0.2	4.36	11.34	434.8 ± 3.5	2.3 ± 0.02
	Person	86.0 ± 0.2	73.9 ± 0.2	83.0 ± 0.2	45.2 ± 0.2				
	Bicycle	88.7 ± 0.2	80.4 ± 0.2	89.2 ± 0.2	58.3 ± 0.2				
	Car	81.1 ± 0.3	64.7 ± 0.3	75.8 ± 0.3	36.1 ± 0.3				
Dual-stream YOLOvXn	All	83.4 ± 0.3	74.2 ± 0.3	82.4 ± 0.3	45.9 ± 0.3	3.38	9.33	500.0 ± 4.1	2.0 ± 0.02
	Person	84.5 ± 0.3	75.1 ± 0.3	83.3 ± 0.3	44.2 ± 0.3				
	Bicycle	85.6 ± 0.3	80.6 ± 0.3	88.6 ± 0.3	58.1 ± 0.3				
	Car	80.1 ± 0.4	66.9 ± 0.4	75.2 ± 0.4	35.5 ± 0.4				
Dual-stream YOLOv10n	All	82.6 ± 0.3	72.1 ± 0.3	81.2 ± 0.3	45.1 ± 0.3	3.73	11.23	666.7 ± 5.3	1.5 ± 0.01
	Person	83.6 ± 0.3	72.4 ± 0.3	82.0 ± 0.3	44.2 ± 0.3				
	Bicycle	85.5 ± 0.3	78.5 ± 0.3	87.3 ± 0.3	57.4 ± 0.3				
	Car	78.6 ± 0.4	65.5 ± 0.4	74.3 ± 0.4	33.6 ± 0.4				
Dual-stream YOLOv11n	All	83.2 ± 0.2	74.2 ± 0.2	81.8 ± 0.2	45.6 ± 0.2	3.79	9.31	370.4 ± 3.0	2.7 ± 0.02
	Person	83.6 ± 0.2	74.8 ± 0.2	83.2 ± 0.2	44.8 ± 0.2				
	Bicycle	85.7 ± 0.2	79.7 ± 0.2	88.2 ± 0.2	57.7 ± 0.2				
	Car	80.2 ± 0.3	68.1 ± 0.3	74.1 ± 0.3	34.4 ± 0.3				
MBNet	All	82.8 ± 0.3	72.5 ± 0.3	80.6 ± 0.3	44.9 ± 0.3	6.8	16.2	285.7 ± 2.5	3.5 ± 0.03
	Person	83.9 ± 0.3	73.2 ± 0.3	81.5 ± 0.3	43.6 ± 0.3				
	Bicycle	86.2 ± 0.3	78.9 ± 0.3	86.8 ± 0.3	57.0 ± 0.3				
	Car	78.3 ± 0.4	65.4 ± 0.4	73.5 ± 0.4	34.1 ± 0.4				
ProbEn	All	83.5 ± 0.2	73.3 ± 0.2	82.1 ± 0.2	45.4 ± 0.2	7.5	17.4	263.2 ± 2.2	3.8 ± 0.03
	Person	84.6 ± 0.2	74.0 ± 0.2	82.8 ± 0.2	44.0 ± 0.2				
	Bicycle	86.9 ± 0.2	79.8 ± 0.2	88.4 ± 0.2	57.6 ± 0.2				
	Car	79.0 ± 0.3	66.1 ± 0.3	75.0 ± 0.3	34.6 ± 0.3				
Dual-stream RT-DETR	All	82.2 ± 0.4	71.6 ± 0.4	81.0 ± 0.4	44.5 ± 0.4	66.34	194.03	70.4 ± 0.9	14.2 ± 0.15
	Person	82.8 ± 0.4	72.1 ± 0.4	81.7 ± 0.4	43.2 ± 0.4				
	Bicycle	84.4 ± 0.4	77.5 ± 0.4	86.6 ± 0.4	55.9 ± 0.4				
	Car	79.4 ± 0.5	65.2 ± 0.5	74.6 ± 0.5	34.5 ± 0.5				
Proposed	All	84.5 ± 0.2	74.2 ± 0.2	82.8 ± 0.2 *	46.5 ± 0.2	3.44	7.58	476.2 ± 3.5	2.1 ± 0.02
	Person	85.8 ± 0.2 *	75.3 ± 0.2	83.8 ± 0.2 *	45.2 ± 0.2				
	Bicycle	88.6 ± 0.2 *	80.5 ± 0.2	89.2 ± 0.2 *	58.3 ± 0.2				
	Car	80.2 ± 0.3	67.0 ± 0.3	75.5 ± 0.3	36.0 ± 0.3				

* Indicates *p* < 0.05 vs. Dual-stream YOLOv11n for the corresponding metric.

**Table 6:** Complexity comparison among the original single-stream YOLOv11n, the dual-stream YOLOv11n baseline, and the proposed method.

Model	Stream Setting	GFLOPs
Original YOLOv11n	Single-stream	4.67
Dual-stream YOLOv11n baseline	Dual-stream	9.31
Proposed	Dual-stream	7.58

**Table 7:** Quantitative comparison of detection accuracy and efficiency among state-of-the-art methods and our proposed model on the M3FD dataset.

Method	Classes	Precision (%)	Recall (%)	mAP@50 (%)	mAP@50:95 (%)	Params (M)	GFLOPs (G)	FPS	Latency (ms)
Dual-stream YOLOv5n	All	77.8 ± 0.4	62.0 ± 0.4	68.5 ± 0.4	43.5 ± 0.4	3.67	9.81	400.0 ± 3.2	2.5 ± 0.02
	Person	78.8 ± 0.4	70.5 ± 0.4	78.3 ± 0.4	45.5 ± 0.4				
	Car	86.5 ± 0.4	77.0 ± 0.4	84.1 ± 0.4	57.0 ± 0.4				
	Bus	84.5 ± 0.5	76.4 ± 0.5	80.6 ± 0.5	61.0 ± 0.5				
	Motorcycle	69.9 ± 0.5	47.5 ± 0.5	52.9 ± 0.5	31.3 ± 0.5				
	Lamp	72.4 ± 0.6	37.3 ± 0.6	45.4 ± 0.6	20.3 ± 0.6				
	Truck	74.6 ± 0.5	63.0 ± 0.5	70.0 ± 0.5	45.8 ± 0.5				
Dual-stream YOLOv8n	All	82.0 ± 0.3	62.8 ± 0.3	70.3 ± 0.3	44.7 ± 0.3	4.36	11.34	270.3 ± 2.8	3.7 ± 0.03
	Person	80.9 ± 0.3	72.5 ± 0.3	78.6 ± 0.3	46.6 ± 0.3				
	Car	87.3 ± 0.3	77.5 ± 0.3	85.1 ± 0.3	58.3 ± 0.3				
	Bus	85.3 ± 0.4	74.4 ± 0.4	78.0 ± 0.4	61.0 ± 0.4				
	Motorcycle	80.7 ± 0.4	45.8 ± 0.4	54.1 ± 0.4	30.4 ± 0.4				
	Lamp	80.8 ± 0.5	37.8 ± 0.5	50.0 ± 0.5	23.4 ± 0.5				
	Truck	76.7 ± 0.4	68.9 ± 0.4	76.0 ± 0.4	48.5 ± 0.4				
Dual-stream YOLOvXn	All	79.5 ± 0.4	62.1 ± 0.4	69.0 ± 0.4	43.8 ± 0.4	3.38	9.34	370.4 ± 3.1	2.7 ± 0.02
	Person	81.1 ± 0.4	70.4 ± 0.4	78.4 ± 0.4	46.0 ± 0.4				
	Car	86.2 ± 0.4	76.5 ± 0.4	84.2 ± 0.4	57.3 ± 0.4				
	Bus	86.0 ± 0.5	75.0 ± 0.5	78.8 ± 0.5	59.5 ± 0.5				
	Motorcycle	73.2 ± 0.5	49.3 ± 0.5	53.5 ± 0.5	32.3 ± 0.5				
	Lamp	73.2 ± 0.6	35.5 ± 0.6	45.9 ± 0.6	21.2 ± 0.6				
	Truck	77.1 ± 0.5	66.2 ± 0.5	73.0 ± 0.5	45.9 ± 0.5				
Dual-stream YOLOv10n	All	78.6 ± 0.4	59.7 ± 0.4	67.8 ± 0.4	42.7 ± 0.4	3.73	11.23	454.5 ± 3.8	2.2 ± 0.02
	Person	79.7 ± 0.4	69.3 ± 0.4	77.5 ± 0.4	45.0 ± 0.4				
	Car	85.5 ± 0.4	74.1 ± 0.4	83.4 ± 0.4	56.3 ± 0.4				
	Bus	81.2 ± 0.5	73.7 ± 0.5	77.1 ± 0.5	58.6 ± 0.5				
	Motorcycle	78.0 ± 0.5	42.2 ± 0.5	52.9 ± 0.5	28.9 ± 0.5				
	Lamp	70.4 ± 0.6	37.1 ± 0.6	46.1 ± 0.6	21.4 ± 0.6				
	Truck	76.7 ± 0.5	61.7 ± 0.5	70.0 ± 0.5	45.4 ± 0.5				
Dual-stream YOLOv11n	All	82.6 ± 0.3	57.5 ± 0.3	66.5 ± 0.3	42.6 ± 0.3	3.79	9.31	344.8 ± 3.0	2.9 ± 0.02
	Person	82.9 ± 0.3	66.9 ± 0.3	77.2 ± 0.3	45.4 ± 0.3				
	Car	88.6 ± 0.3	74.6 ± 0.3	83.9 ± 0.3	57.4 ± 0.3				
	Bus	84.3 ± 0.4	74.4 ± 0.4	78.3 ± 0.4	59.3 ± 0.4				
	Motorcycle	81.2 ± 0.4	37.9 ± 0.4	47.6 ± 0.4	28.6 ± 0.4				
	Lamp	79.8 ± 0.5	31.1 ± 0.5	42.8 ± 0.5	19.7 ± 0.5				
	Truck	78.7 ± 0.4	60.3 ± 0.4	69.3 ± 0.4	45.4 ± 0.4				
MBNet	All	80.0 ± 0.4	61.2 ± 0.4	68.2 ± 0.4	43.0 ± 0.4	6.8	16.2	285.7 ± 2.7	3.5 ± 0.03
	Person	80.5 ± 0.4	70.7 ± 0.4	77.9 ± 0.4	45.5 ± 0.4				
	Car	85.8 ± 0.4	75.6 ± 0.4	83.6 ± 0.4	56.5 ± 0.4				
	Bus	83.4 ± 0.5	73.9 ± 0.5	77.5 ± 0.5	59.0 ± 0.5				
	Motorcycle	76.1 ± 0.5	44.5 ± 0.5	51.6 ± 0.5	29.5 ± 0.5				
	Lamp	73.9 ± 0.6	35.6 ± 0.6	44.8 ± 0.6	20.4 ± 0.6				
	Truck	75.5 ± 0.5	63.8 ± 0.5	71.2 ± 0.5	45.6 ± 0.5				
ProbEn	All	80.8 ± 0.3	61.7 ± 0.3	68.8 ± 0.3	43.3 ± 0.3	7.5	17.4	263.2 ± 2.4	3.8 ± 0.03
	Person	81.2 ± 0.3	71.0 ± 0.3	78.2 ± 0.3	45.8 ± 0.3				
	Car	86.6 ± 0.3	75.9 ± 0.3	84.0 ± 0.3	56.8 ± 0.3				
	Bus	83.9 ± 0.4	74.5 ± 0.4	78.2 ± 0.4	59.5 ± 0.4				
	Motorcycle	77.5 ± 0.4	45.0 ± 0.4	52.1 ± 0.4	30.0 ± 0.4				
	Lamp	74.8 ± 0.5	36.2 ± 0.5	45.5 ± 0.5	20.8 ± 0.5				
	Truck	76.0 ± 0.4	64.6 ± 0.4	71.7 ± 0.4	46.0 ± 0.4				
Dual-stream RT-DETR	All	79.2 ± 0.5	60.5 ± 0.5	67.2 ± 0.5	42.2 ± 0.5	66.34	194.03	69.9 ± 1.0	14.3 ± 0.15
	Person	79.8 ± 0.5	69.9 ± 0.5	77.0 ± 0.5	44.8 ± 0.5				
	Car	84.9 ± 0.5	74.5 ± 0.5	82.6 ± 0.5	55.6 ± 0.5				
	Bus	82.6 ± 0.6	73.2 ± 0.6	76.6 ± 0.6	58.1 ± 0.6				
	Motorcycle	74.8 ± 0.6	43.3 ± 0.6	50.5 ± 0.6	28.4 ± 0.6				
	Lamp	72.9 ± 0.7	34.5 ± 0.7	43.9 ± 0.7	19.7 ± 0.7				
	Truck	74.5 ± 0.6	62.9 ± 0.6	70.2 ± 0.6	44.9 ± 0.6				
Proposed	All	79.2 ± 0.3	61.8 ± 0.3	69.0 ± 0.3 *	43.8 ± 0.3	3.44	7.58	384.6 ± 3.2	2.6 ± 0.02
	Person	80.5 ± 0.3	71.2 ± 0.3	78.8 ± 0.3 *	46.0 ± 0.3				
	Car	86.2 ± 0.3	76.5 ± 0.3	84.1 ± 0.3	57.0 ± 0.3				
	Bus	85.0 ± 0.4	75.9 ± 0.4	80.2 ± 0.4 *	60.5 ± 0.4				
	Motorcycle	74.8 ± 0.4	46.5 ± 0.4	53.5 ± 0.4	31.0 ± 0.4				
	Lamp	75.3 ± 0.5	36.2 ± 0.5	46.0 ± 0.5	20.8 ± 0.5				
	Truck	76.5 ± 0.4	65.8 ± 0.4	72.1 ± 0.4 *	47.2 ± 0.4 *				

* Indicates *p* < 0.05 vs. YOLOv11n for the corresponding metric.

**Table 8:** Edge-device inference performance of representative methods on Jetson Nano *.

Method	Input Size	Backend	Precision Mode	Params (M)	GFLOPs (G)	Latency (ms)	FPS
Dual-stream YOLOv5n	640 × 640	TensorRT	FP16	3.67	9.81	45.2 ± 0.3	22.1 ± 0.2
Dual-stream YOLOv8n				4.36	11.34	52.6 ± 0.4	19.0 ± 0.2
Dual-stream YOLOvXn				3.38	9.34	44.1 ± 0.3	22.7 ± 0.2
Dual-stream YOLOv10n				3.73	11.23	51.3 ± 0.4	19.5 ± 0.2
Dual-stream YOLOv11n				3.79	9.31	43.5 ± 0.3	23.0 ± 0.2
MBNet				6.8	16.2	71.4 ± 0.5	14.0 ± 0.2
ProbEn				7.5	17.4	76.9 ± 0.6	13.0 ± 0.2
Dual-stream RT-DETR				66.34	194.03	N/A	N/A
Proposed				3.44	7.58	38.5 ± 0.3	26.0 ± 0.2

* All experiments were performed on the M3FD test set on Jetson Nano with batch size = 1 and input size = 640 × 640. TensorRT with FP16 inference was used for all methods. Latency and FPS were measured after warm-up under the same deployment setting.

**Table 9:** Ablation study of the proposed modules on the FLIR and M3FD datasets. The baseline is a dual-stream YOLOv11n with naive concatenation fusion.

Method	GFM	SPD-Conv	Ghost-Neck	Params(M)	GFLOPs (G)	FLIR		M3FD	
						mAP@50(%)	mAP@50:95(%)	mAP@50(%)	mAP@50:95(%)
Dual-stream baseline				3.79	9.31	81.9 ± 0.2	45.6 ± 0.2	66.5 ± 0.3	42.6 ± 0.3
+GFM	✓			3.82	9.38	82.3 ± 0.2	45.9 ± 0.2	68.0 ± 0.3	43.2 ± 0.3
+SPD-Conv	✓	✓		3.75	9.12	83.1 ± 0.2	46.8 ± 0.2	69.4 ± 0.3	44.3 ± 0.3
Proposed	✓	✓	✓	3.44	7.58	82.8 ± 0.2	46.5 ± 0.2	69.0 ± 0.3	43.8 ± 0.3

**Table 10:** Isolated contribution of GFM under the same dual-stream framework.

Model Variant	Fusion Strategy	Params(M)	GFLOPs (G)	FLIR		M3FD	
				mAP@50(%)	mAP@50:95(%)	mAP@50(%)	mAP@50:95(%)
Dual-stream baseline	Concatenation	3.79	9.31	82.2	45.9	66.8	42.9
Dual-stream baseline + GFM	Global-context gating	3.82	9.38	82.5	46.1	68.2	43.4

**Table 11:** Effect of SPD-Conv on scale-wise AP_s, AP_m, and AP_l on the FLIR and M3FD datasets under the MS-COCO size protocol *.

Variant	FLIR			M3FD		
	AP_s	AP_m	AP_l	AP_s	AP_m	AP_l
w/o SPD-Conv	31.8	55.9	60.7	16.3	54.1	73.4
w/SPD-Conv	34.2	56.5	61.2	18.5	54.8	73.9
Gain	+2.4	+0.6	+0.5	+2.2	+0.7	+0.5

* “w/o SPD-Conv” denotes the baseline variant without the SPD-Conv module, while “w/SPD-Conv” denotes the complete model with SPD-Conv. “Gain” indicates the absolute improvement introduced by SPD-Conv. AP_S, AP_M, and AP_L denote the average precision under the MS-COCO AP@0.50:0.95 evaluation protocol for small $\left(area<32^{2}\right)$, medium $\left(32^{2}\leq area<96^{2}\right)$, and large $\left(area\geq 96^{2}\right)$ objects, respectively.

**Table 12:** Scale-wise AP comparison of representative methods on the FLIR and M3FD datasets under the MS-COCO size protocol *.

Method	FLIR			M3FD		
	AP_s	AP_m	AP_l	AP_s	AP_m	AP_l
YOLOv5n	33.6 ± 0.3	51.5 ± 0.4	53.4 ± 0.5	17.1 ± 0.2	53.2 ± 0.3	73.0 ± 0.4
YOLOv8n	33.2 ± 0.3	57.6 ± 0.4	69.9 ± 0.5	16.6 ± 0.2	54.1 ± 0.3	71.4 ± 0.4
YOLOvXn	32.8 ± 0.3	56.8 ± 0.4	59.5 ± 0.5	16.6 ± 0.2	53.7 ± 0.3	72.8 ± 0.4
YOLOv10n	33.0 ± 0.3	54.5 ± 0.4	65.7 ± 0.5	17.4 ± 0.2	53.3 ± 0.3	72.3 ± 0.4
YOLOv11n	32.3 ± 0.3	55.2 ± 0.4	59.6 ± 0.5	17.4 ± 0.2	53.2 ± 0.3	72.2 ± 0.4
MBNet	32.2 ± 0.3	53.7 ± 0.4	58.2 ± 0.5	17.3 ± 0.2	52.7 ± 0.3	71.6 ± 0.4
ProbEn	32.6 ± 0.3	55.0 ± 0.4	59.5 ± 0.5	17.6 ± 0.2	53.0 ± 0.3	72.1 ± 0.4
RT-DETR	30.6 ± 0.4	51.5 ± 0.5	53.4 ± 0.6	16.6 ± 0.3	45.4 ± 0.4	63.0 ± 0.5
Proposed	34.2 ± 0.2	56.5 ± 0.3	61.2 ± 0.4	18.5 ± 0.2	54.8 ± 0.3	73.9 ± 0.4

* AP_s, AP_m, and AP_l denote the average precision under the MS-COCO AP@0.50:0.95 evaluation protocol for small $\left(area<32^{2}\right)$, medium $\left(32^{2}\leq area<96^{2}\right)$, and large $\left(area\geq 96^{2}\right)$ objects, respectively.

## Data Availability

The original contributions presented in this study are included in the article. Further inquiries can be directed to the corresponding author.
